# Re-examining extreme carbon isotope fractionation in the coccolithophore *Ochrosphaera neapolitana*

**DOI:** 10.1038/s41467-022-35109-4

**Published:** 2022-12-12

**Authors:** Hongrui Zhang, Ismael Torres-Romero, Heather M. Stoll

**Affiliations:** Geological Institute, ETH Zürich, 8092 Zürich, Switzerland

**Keywords:** Biogeochemistry, Marine biology, Carbon cycle

**arising from** Y.-W. Liu et al. *Nature Communications* 10.1038/s41467-018-04463-7 (2018)

In coccolithophores, stable isotopes recorded in both the calcite exoskeleton (coccoliths), and organic carbon (C_org_), can reflect their physiological response to environment, and thereby have a wide usage in paleoclimate and biogeochemistry studies. Recently, Liu et al.^1^ reported that coccolithophore *Ochrosphaera neapolitana* has much more positive carbon isotope fractionations relative to dissolved inorganic carbon (DIC) in both coccolith and C_org_ compared with those published previously for other species^2–4^ and attributed such unexpected positive carbon isotope fractionations to a unique carbon pathway in this species. However, we find that these extreme isotopic fractionations should be attributed to the poor constraints in DIC carbon isotope ratios instead of the coccolithophores’ physiological response to *p*CO_2_. More careful measurements of DIC carbon isotope would benefit data interpretations and comparisons in future laboratory culture works focusing on phytoplankton’s response to ocean acidification.

In order to study this unusual isotopic fractionation, we carried out another independent culture of *O. neapolitana* under a low CO_2_ environment (*p*CO_2_ ≈ 254ppm and CO_2_(aq) ≈ 8.7 μM). The results of carbon isotope fractionation show no exaggerated positive values in neither coccolith nor C_org_ (Fig. 1). The carbon isotope fractionation between coccolith and DIC (Δ^13^C_coccolith-DIC_ = δ^13^C_coccolith_ − δ^13^C_DIC_) is −1.86 ± 0.43‰ (standard deviation of three biological replicates) and that of C_org_ (Δ^13^C_Corg-DIC_ = δ^13^C_Corg_ − δ^13^C_DIC_) is −23.14 ± 0.57‰, which are in the same range as published carbon isotope fractionations of other species.

**Fig. 1:**
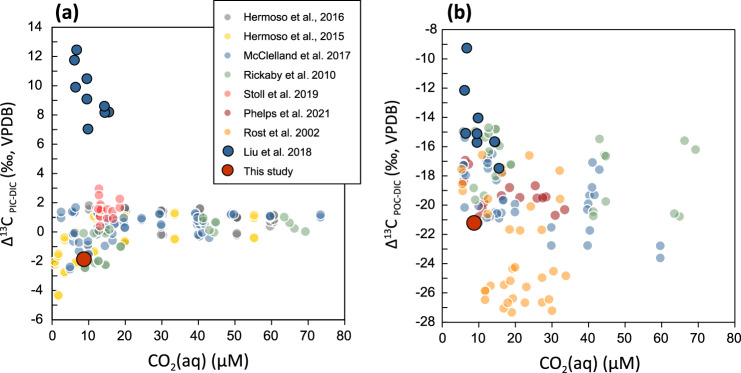
Carbon isotope fractionations (Δ^13^C) of coccolithophores in different laboratory culture studies. **a** Carbon isotope fractionation between PIC (particulate inorganic carbon from harvested cells) and DIC (dissolved inorganic carbon from seawater). **b** Carbon isotope fractionation between POC (particulate organic carbon from harvested cells) and DIC. Markers plotted in red and blue are *Ochrosphaera neapolitana* in this study and in Liu et al.^1^, respectively. Markers in other colors are results from other publications^2–4, 12–15^ using different species of coccolithophores including *Coccolithus pelagicus*, *Gephyrocapsa oceanica*, *Calcidiscus leptoporus*, and *Emiliania huxleyi*.

We propose that the extreme positive carbon isotope fractionations and fractionation trend with *p*CO_2_, originally reported by Liu et al.^1^ might be caused by inaccurate estimation of the DIC carbon isotope ratio (δ^13^C_DIC_). Instead of directly measuring δ^13^C_DIC_ during culture, the authors assumed that δ^13^C_DIC_ reached equilibrium with CO_2_ sources through the whole experiment. However, in reality, the δ^13^C_DIC_ could be positively shifted by two processes, (1) the isotopic disequilibrium between CO_2_(g) and DIC during bubbling and (2) the selective uptake of light carbon by algae photosynthesis during culture.

As previously suggested, seawater culture medium should be bubbled at least overnight to achieve a stable carbonate system^5^. Yet, the isotopic equilibrium is much slower than the chemical equilibrium^6^, because the carbon atoms need to be fully exchanged between gas phase and liquid phase before reaching an isotopic equilibrium. Moreover, all culture experiments in Liu et al.^1^ were carried out using 38 L glass aquaria, thus increasing the time for such a large DIC pool to reach isotopic equilibrium with the gas phase. In Liu et al.^1^, the details in aeration process, such as aeration time and initial δ^13^C_DIC_, were not described. Based on the final *p*CO_2_ in all treatments being lower than the target *p*CO_2_ by at least 20%, we infer that the seawater media were pre-bubbled before the incubation of coccolithophores and that there was no further CO_2_ aeration during the culture. To evaluate the extent of isotopic disequilibrium in the pre-bubbling process, here we carry out isotopic simulations to trace the carbon atom exchanging process between DIC and CO_2_ source (more details in Methods and Supplementary Note 1).

One key parameter in the simulations is the carbon atom exchanging rate constant between CO_2_(g) and CO_2_(aq) (*k*_E_). In our previous work, we measured this constant in two photobioreactor systems featuring aeration with specified CO_2_ concentration; this constant ranged from 3.4 × 10^−5^ to 8.7 × 10^−5^ mol s^−1^ atm^−1^ ^7^. We conduct simulations using *k*_E_ ranging from 10^−4^ to 10^−3^ mol s^−1^ atm^−1^. For reproducing the DIC carbon isotope evolution in Liu et al.^1^, a *k*_E_ of ~3.6 × 10^−4^ is more realistic considering their gas flux was 1.5 L min^−1^, 6 times as our bubbling system in ETH Zurich. Another important parameter is the initial carbon isotope ratio difference between DIC and CO_2_ source. Based on equilibrium δ^13^C_DIC_ described by Liu et al.^1^, we back-calculated the isotopic signatures of the CO_2_ sources employed among treatments in Liu et al.^1^. These range from −14‰ to −38‰ (Supplementary Table S1). A larger initial difference can potentially cause a more significant disequilibrium between DIC and CO_2_(g) leading to an underestimation of δ^13^C_DIC_ (as simulated in Fig. 2) and thereby more positive coccolith and C_org_ carbon isotope fractionations. For example, the carbon isotope disequilibrium in ‘280 ppm’ treatment could be larger than the disequilibrium in other two treatments by up to 8‰ even after two weeks aeration (green line in Fig. 2a). Hence, we infer that the isotopic disequilibrium between gas phase and liquid phase during the pre-bubbling process is the main reason why the calculated carbon isotope fractionations in ‘280 ppm’ treatment were much more positive than the results in the other two treatments, instead of the lower *p*CO_2_.

**Fig. 2:**
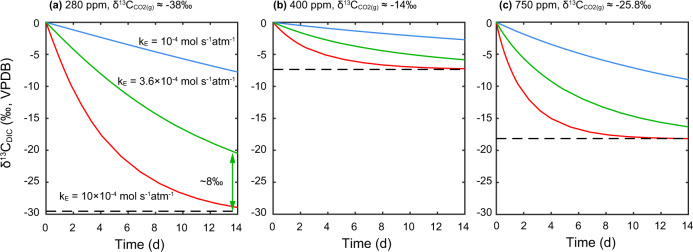
Simulated DIC carbon isotope ratios evolution during bubbling. Panel a, b, c are simulations for different *p*CO2 and initial δ^13^C_DIC_. Blue curves: *k*_E_ = 10^−4^ mol s^−1^ atm^−1^ (a slower equilibrium). Red curves: *k*_E_ = 10^−3^ mol s^−1^ atm^−1^ (a faster equilibrium). Green curves: *k*_E_ = 3.6 × 10^−4^ mol s^−1^ atm^−1^ (potential exchanging rate for Liu et al.^1^). The black dashed lines represent the isotopic equilibrium δ^13^C_DIC_. The disequilibrium between DIC and gas in 280 ppm treatment after two weeks of bubbling is marked by the vertical green arrows.

Photosynthesis preferentially takes up the light carbon leading to a positive shift of remaining DIC. Ignoring the carbon isotope effect of calcification (much smaller effect compared with photosynthesis), this positive shift in DIC carbon isotope ratio can be estimated by mass balance (Supplementary Note 3). Given a cell density of 10^5^ cell mL^−1^ as in Liu et al.^1^, the carbon isotope fractionation could be as large as +3‰. We suggest that this DIC carbon isotope shift during culture can explain ~20% of the abnormal carbon isotope fractionation in Liu et al.^1^, especially the large carbon isotope differences among replicates within the same treatment.

Our culture data and simulations show evidence that the carbon isotope fractionation results in Liu et al.^1^ could have a significant bias due to the lack of direct δ^13^C_DIC_ measurements. Despite acknowledging their values might have an offset (the absolute values on fractionation could differ from real values), they still claimed that the trend of carbon isotope fractionation on *p*CO_2_ should be robust. Nevertheless, such trend in Fig. 3 of Liu et al.^1^ would already not be significant when we account for the different disequilibrium offsets among the three treatments. Indeed, simulations of δ^13^C_DIC_ evolution suggest that the offset in ‘280 ppm’ treatment could be as large as 10‰ more positive than the other two treatments. Thus, their measurements do not support their conclusion that the calcification and photosynthesis of *O. neapolitana* share the same carbon pool.

Carbon isotope techniques in laboratory cultures have been widely used to trace the ocean acidification effects on phytoplankton, and thereby calibrate a robust *p*CO_2_ proxy in the paleo-climate field. Considering the importance of culture studies with isotopic measurement, cautions should be exercised in experiment design. First, a larger volume of culture medium entails a longer isotopic equilibrium time during the CO_2_ aeration process, which should be noted for future work especially using bubbling methods. More importantly, the δ^13^C_DIC_ should be measured directly to assess fractionation correctly in future isotopic studies. Only measuring the DIC carbon isotope at the beginning of culture is not enough for the batch cultures, because the positive shifts of DIC carbon isotope due to photosynthesis could be as large as 3‰. By following the recommendations described here, we can make the isotopic results comparable and help to better understand the phytoplankton’s response to ocean acidification.

## Methods

### Laboratory culture

*Ochrosphaera neapolitana* (RCC1357) was precultured in K/2 medium without Tris buffer^8^ using artificial seawater (ASW) supplemented with NaHCO_3_ and HCl to yield an initial DIC of 2050 µM. In triplicate, 1-L bottles were filled with 150 mL of seawater medium with air in the bottle headspace and inoculated with a mid-log phase preculture at an initial cell concentration of 10^4^ cells mL^−1^. Cultures were grown at 18 °C under a warm white LED light at 100 ± 20 µE on a 16h-light/8h-dark cycle. Bottles were orbitally shaken at 60 rpm to keep cells in suspension. Cell growth was monitored with a Multisizer 4e particle counter and sizer (Beckman Coulter). At ~1.4 × 10^5^ cells mL^−1^, cells were diluted up to 300 mL to 2–3 × 10^4^ cells mL^−1^ and harvested after 2 days of more exponential growth up to 7.9 ± 0.6 × 10^4^ cells mL^−1^. More detailed culture results are listed in the Supplementary Note 1.

Immediately after harvesting, pH was measured using a pH probe calibrated with Mettler Toledo NBS standards (it should be noted here that high ionic strength calibration standards would be optimal for pH measurement of liquids like seawater). There was a carbonate system shift during the batch culture and more details are shown in Supplementary Fig. S1. Cells in 50 mL were pelleted by centrifuging at ~1650 × *g* for 5 min. Seawater supernatant was analyzed for DIC and δ^13^C_DIC_ by injecting 3.5 mL into an Apollo analyzer and injecting 1 mL into He-flushed glass vials containing H_3_PO_4_ for the Gas Bench.

For seawater DIC, an Apollo SciTech DIC-C13 Analyzer coupled to a Picarro CO_2_ analyzer was calibrated with in-house NaHCO_3_ standards dissolved in deionized water at different known concentrations and δ^13^C values from −4.66 to −7.94‰. δ^13^C_DIC_ in media were measured with a Gas Bench II with an autosampler (CTC Analytics AG, Switzerland) coupled to ConFlow IV Interface and a Delta V Plus mass spectrometer (Thermo Fischer Scientific). Pelleted cells were snap-frozen with N_2_ (l) and stored at −80 °C. For PIC analysis, pellet was resuspended in 1 mL methanol and vortexed. After centrifugation, the methanol phase with extracted organics was removed and the pellet containing the coccoliths was dried at 60 °C overnight. About 300 mg of dried coccolith powder were placed in air-tight glass vials, flushed with He and reacted with five drops of phosphoric acid at 70 °C. PIC δ^13^C and δ^18^O were measured by the same Gas Bench system. The system and abovementioned in-house standards were calibrated using international standards NBS 18 (δ^13^C = −5.01‰, δ^18^O = +23.00‰) and NBS 19 (δ^13^C = +1.95‰, δ^18^O = +2.2‰). The analytical error for DIC concentration and δ^13^C is <10 μM and 0.1‰, respectively.

POC and PON were determined from cells harvested on pre-combusted QFF filters and deep-frozen until analysis. Inorganic carbon from cells on filters was removed by fuming sulfurous acid during 24 h. Filters were placed inside a desiccator on a porous tray and 50 mL sulfurous acid below was fumed with a vacuum pump. Gases were evacuated and filters were further dried at 60 °C overnight. Right before Elemental Analysis (EA), filters were compacted and wrapped into tin cups with the help of tweezers and a press. Samples loaded on a 96-well plate were combusted in the oxidation column at 1020 °C of a Thermo Fisher Flash-EA 1112 coupled with a Conflo IV interface to a Thermo Fisher Delta V-IRMS (isotope ratio mass spectrometer). Combustion gas passed through a reduction column at 650 °C producing N_2_ and CO_2_ which were separated by chromatography and into a split to the IRMS for an on-line isotope measurement.

### Simulations of carbon isotope evolution during aeration

The DIC carbon isotope evolution model is simplified from the model in Zhang et al.^7^ The exchanging rate (with a unit of mol s^−1^) between CO_2_(g) and CO_2_(aq) depends on the CO_2_ gradient and exchanging rate constant (*k*_E_, with a unit of ppm s^−1^): ER =  *k*_E_(CO_2(*g*)_–*k*_*H*_CO_2(aq)_), where the *k*_H_ is Henry’s law constant with a unit of ppm µM^−1^. The evolutions of DIC and DIC carbon isotope ratios during CO_2_ aeration can be calculated by four differential equations:

1 $$\frac{{d{{{{{\mathrm{C}}}}}}}}{{dt}}=\,\frac{{k}_{{{{{{\mathrm{E}}}}}}}}{V}\left({{{{{\mathrm{G}}}}}}-{{{{{\mathrm{C}}}}}}\,{k}_{{{{{\mathrm{{H}}}}}}}\right)+\left({k}_{-1}{{{{{{\mathrm{H}}}}}}}^{+}+{k}_{-4}\right){{{{{\mathrm{C}}}}}}-\left({k}_{+1}+{k}_{+4}\,{{{{{{{\mathrm{OH}}}}}}}}^{-}\right)\,{{{{{\mathrm{B}}}}}} \; \; {{{{{{\mathrm{XB}}}}}}}1$$

2 $$\frac{{d}^{13}{{{{{\mathrm{C}}}}}}}{dt}=\frac{{k}_{{{{{{\mathrm{E}}}}}}}}{V}\left({\,}^{13}{{{{{\mathrm{G}}}}}}\,{\alpha }_{g2aq}-{\,}^{13}{{{{{\mathrm{C}}}}}}\,{k}_{{{{{{\mathrm{H}}}}}}}{\alpha }_{aq2g}\right)+\left({k}_{+1}^{13}+{k}_{+4}^{13}{{{{{\mathrm{O}}}}}}{{{{{{\mathrm{H}}}}}}}^{-}\right){\,}^{13}{{{{{\mathrm{B}}}}}}\,{{{{{{\mathrm{X}}}}}}}^{13}{{{{{\mathrm{B1}}}}}}-\left({k}_{-1}^{13}\,{{{{{{\mathrm{H}}}}}}}^{+}+{k}_{-4}^{13}\right){\,}^{13}{{{{{\mathrm{C}}}}}}$$

3 $$\frac{{d{{{{{\mathrm{B}}}}}}}}{{dt}}=-\,\left({k}_{-1}{{{{{{\mathrm{H}}}}}}}^{+}+{k}_{-4}\,\right){{{{{\mathrm{C}}}}}}+\left({k}_{+1}+\,{k}_{+4}\,{{{{{{{\mathrm{OH}}}}}}}}^{-}\right)\,{{{{{\mathrm{B}}}}}} \; \; {{{{{{\mathrm{XB}}}}}}}1$$

4 $$\frac{{d}^{13}{{{{{\mathrm{B}}}}}}}{dt}=-\left({k}_{-1}^{13}{{{{{{\mathrm{H}}}}}}}^{+}+{k}_{-4}^{13}\right){\,\!}^{13}{{{{{\mathrm{C}}}}}}+\left({k}_{+1}^{13}+{k}_{+4}^{13}\,{{{{{\mathrm{O}}}}}}{{{{{{\mathrm{H}}}}}}}^{-}\right){\,\!}^{13}{{{{{\mathrm{B}}}}}} \; \; {{{{{{\mathrm{X}}}}}}}^{13}{{{{{\mathrm{B}}}}}}1$$

where capital letters G, C, B, H, and OH represent CO_2_(g), CO_2_(aq), HCO_3_^−^ + CO_3_^2−^, H^+^, and OH^−^, respectively. The V stands for volume. The *α* is the isotopic fractionation, e.g. *α*_g2aq_ represents the carbon isotope fractionation of CO_2_ gas diffusion into liquid phase. The XB1 and X^13^B1 are the fraction of HCO_3_^−^ in (HCO_3_^−^ + CO_3_^2−^) and the fraction of H^13^CO_3_^−^ in (H^13^CO_3_^−^ + ^13^CO_3_^2−^). The XB1 can be calculated by ${{{{{\rm{XB}}}}}}1=\frac{1}{1+\frac{K2}{\left[{{{{{{\mathrm{H}}}}}}}^{+}\right]}}$ and the X^13^B1 can be calculated by ${{{{{{\rm{X}}}}}}}^{13}{{{{{\mathrm{B}}}}}}1=\frac{1}{1+\frac{K2}{\left[{{{{{{\mathrm{H}}}}}}}^{+}\right]}{\alpha }_{{{{{{{\mathrm{CO}}}}}}_{3}}-{{{{{{\mathrm{HCO}}}}}}_{3}}}}$, where the ${\alpha }_{{{{{{{\mathrm{CO}}}}}}}3-{{{{{{\mathrm{HCO}}}}}}}3}$ is the carbon isotope fractionation between CO_3_^2−^ and HCO_3_^−^^9^. The *k*_+1_ is the reaction rate constant of CO_2_ hydration, which can be calculated by ${{{{{\rm{ln}}}}}}{k}_{\!+1}=1246.98-\,\frac{61900}{{T}_{k}}-183{{{{{\rm{ln}}}}}}{T}_{k}$^10^. The *k*_−1_, the reaction rate constant of HCO_3_^−^ dehydration, can be calculated from *k*_+1_, ${k}_{-1}=\,\frac{{k}_{+1}}{K1}$ . The *k*_−4_ and *k*_+4_ are the reaction rate constants of CO_2_ hydroxylation and HCO_3_^−^ dihydroxylation. The *k*_+4_ is calculated by$\,{{{{{\rm{ln}}}}}}{k}_{+4}=17.67- \,\frac{2790.47}{{T}_{k}}$^10^ and ${k}_{-4}={k}_{+4}\frac{{K}_{{{{{{\mathrm{w}}}}}}}}{K1}$, where *K*_w_ is the stoichiometric ion product of water. The *K*1 and *K*2 are the first and second dissociation constants of carbonic acid and in this study, we employed equations from^11^, in which the K1 and K2 were calculated for pH in NBS scale. The reaction rate constants for ^13^C (${k}_{-1}^{13}$, ${k}_{+1}^{13}$, ${k}_{-4}^{13}$ and ${k}_{+4}^{13}$). The initial values for these differential equations are described in the Supplementary Note 2.

## Supplementary information

Supplementary Information

## Data Availability

All culture data generated in this study can be found in the main text and Supplementary Note 1. Source data are provided with this paper.

## Source data

Source Data
