# Supplementary material for: Re-examining extreme carbon isotope fractionation in the coccolithophore Ochrosphaera neapolitana
Source: Nat Commun. 2022 Dec 12;13:7606. doi: 10.1038/s41467-022-35109-4 (PMC9744870; doi:10.1038/s41467-022-35109-4)
Supplement: Supplementary file 1 — Supplementary Information [file 41467_2022_35109_MOESM1_ESM.pdf]

Supplementary information for "Re-examining extreme carbon isotope fractionation in the coccolithophore *Ochrosphaera neapolitana*"

Hongrui Zhang[1]*, Ismael Torres-Romero[1]*, Heather M. Stoll[1]

[1] Geological Institute, ETH Zürich, Sonnegstrasse 5, 8092, Zürich, Switzerland

* These two authors contribute equally to this work. Correspondence and requests for materials should be addressed to H.Z. (zhh@ethz.ch) or I.T. (iromero@ethz.ch).

The PDF files includes:

Supplementary Note 1, 2, 3

Table S1

Figure S1 to S3

Additional References

Supplementary Note 1. Culture results

The average growth rate of *Ochrosphaera neapolitana* in the three replicates is 0.8 d^{-1} (**Figure S1a**). The DIC carbon isotope ratios of culture medium were monitored in the last day before harvest, which show about 1.6 ‰ positive shift mainly due to photosynthesis (red squares in **Figure S1c**). The organic carbon per cell at the end of culture is about 46.7 pg and inorganic carbon per cell is about 4.6 pg resulting a PIC:POC ratio about 0.1.

The final pH of culture medium was 8.5 in NBS scale (8.37 in total scale) and final DIC concentration was ~1520 µM, which indicate a significant carbonate system shift during the culture. To evaluate the pCO_2 shift during the culture, we estimate the DIC and TA evolution by cell growth rate, PIC per cell and POC per cell as following:

$$DIC_t = DIC_f + (PIC + POC)(CD_f - CD_t)$$

$$TA_t = TA_f + 2PIC(CD_f - CD_t)$$

where the CD are the cell density and the subscripts f and t represent end and during culture, respectively. After having the DIC and TA evolutions, the pH, the evolutions of pH, pCO_2 and $CO_{2(aq)}$ are calculated by the matlab version of CO2SYS [1]. We calculate the average pCO_2 by considering more cells were produced in the late stage of culture, resulting an overall average pCO_2 of 254ppm.

Supplementary Note 2. Simulation of isotope evolution in aeration process

In Liu et al (2018), they had three different CO_2 treatments: 280 ppm, 400 ppm and 750 ppm. The carbon source of 280 ppm CO_2 is compressed CO_2 with carbon isotope ratios of -38‰, which is a common value for the commercial CO_2 gas sourcing from fossil fuels with negative carbon isotope signature. The carbon source of 400 ppm CO_2 is compressed air, in which the carbon isotope of CO_2 is about -15‰ (calculated from −6.67‰ of the equilibrium DIC at T=20°C). The 750 ppm CO_2 was mixed from compressed pure CO_2 and compressed air. Based on mass balance, the CO_2 contribution of pure CO_2 and compressed air should be about 1:1.33. Then the carbon isotope of 750 ppm CO_2 should be around -24.8‰, which fits with the carbon isotope of balanced DIC (-14‰) in Liu et al. (2018) considering a 9.2‰ fractionation between DIC and CO_2 at 20°C.

The volume of seawater is 38 L. The DIC concentrations and pH are the same with the carbonate chemistry listed in Liu et al. (2018). The selection of exchanging rate constant between CO_2 gas and DIC (k_E) has been described in the main text. The initial carbon isotope ratio of un-bubbled seawater DIC was not described in Liu et al. (2018). Normally it should be around -6 to 2‰ (from commercial artificial seawater to nature seawater). In the main text **Figure 2**, we show the simulation results using the value of 0‰, and here we illustrate that the carbon isotope disequilibrium with other initial DIC carbon isotope ratios share a similar pattern with each other (**Figure S2**). All initial parameters employed in simulations are listed in **Table S1**.

Supplementary Note 3. Estimate the carbon isotope shifts due to photosynthesis

To perform a simple estimation on the carbon isotopic shift of DIC due to photosynthesis, here we ignore the carbon isotope effect of calcification. Because calcite calcification has a much smaller carbon isotopic fractionation (~1‰) compared with photosynthesis (~15-25‰). Moreover, the PIC:POC ratio of *O. neapolitana* in this study is ~0.1, which means the calcification rate is only 10% of photosynthesis rate. Hence the positive shift in DIC carbon isotope ratio can be estimated by mass balance:

$$C_{org}\ \varepsilon_{pho} + DIC_{res}\ \varepsilon_{DIC} = 0$$

where the C_{org} is the total organic carbon at the end of culture, which can be calculated by cell density multiplied by POC per cell (~46.7 pg per cell in this study). The ε_{pho} is the fractionation of POC to DIC (~23‰ in this study). The DIC_{res} is the amount of residual DIC after culture. The ε_{DIC} is the carbon isotope fractionation of residual DIC to initial DIC, which can be calculated by DIC_{res} and cell density, and is independent of the volume of culture medium. Here we calculate the shift of DIC carbon isotope with the cell density from 10^4 to 10^5 cell mL^{-1}, a range in which cells grow exponentially in diluted cultures, and the DIC concentration covering 200 to 5000 µM (**Figure S3**). The results highlight that increasing cell density sharply shifts DIC towards more positive isotope ratios.

Table S1. Initial values in the simulations

Treatment	Real pCO_2 (ppm)	$\delta^{13}C_{CO_{2(g)}}$ (‰)	k_E (mol s^{-1} ppm^{-1})	Initial $\delta^{13}C_{DIC}$ (‰)	[DIC] (µM)	pH_{NBS}
280 ppm	226	-38	10^{-4}– 10^{-3}	-6 – 2	2414	8.3
400 ppm	338	-15	10^{-4}– 10^{-3}	-6 – 2	2700	8.2
750 ppm	520	-25.8	10^{-4}– 10^{-3}	-6 – 2	2685	8

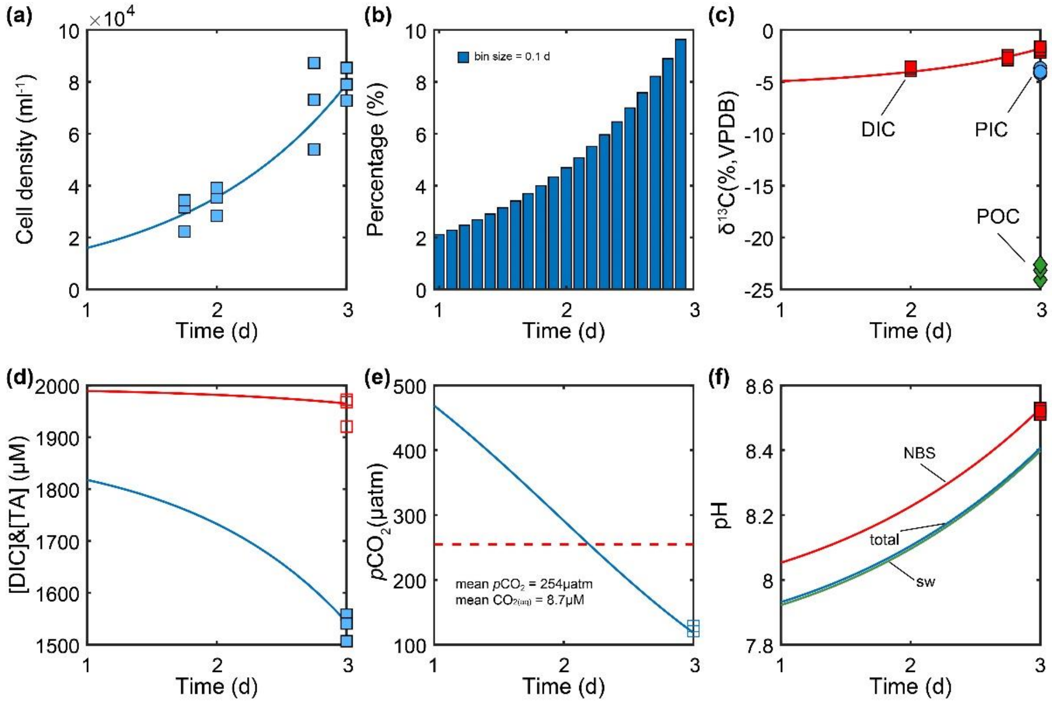

Figure S1. The measurements and simulations of batch culture. **(a)** Cell density. The squares are measurements on particle counter and the line represents the simulated cell density. **(b)** The percentage of cell produced in 0.1 day to total cell harvested at the end of culture. Note that more cells were produced during day 2. **(c)** Carbon isotope ratios of DIC (red curve and squares), PIC (blue dots) and POC (green diamonds). The curve is calculated by simple mass balance and other symbols are measurements. **(d)** Total alkalinity (TA, red) and dissolved inorganic carbon (DIC, blue) concentrations. The solid blue squares are DIC measurements at the end of the culture and red squares are TA calculated by CO2SYS. **(e)** pCO_2 dynamics during the culture. The blue squares are pCO_2 estimated by CO2SYS and blue line is pCO_2 back-calculated by TA and DIC in (d). The red dashed line represents the average pCO_2 cells growing in, which is calculated by a weighted average considering the varying cell production throughout the culture. **(f)** The pH evolutions during the culture. The red squares are measurements at the end of culture. The red, blue and green lines represent NBS (or NIST), total scale and seawater scale, respectively.

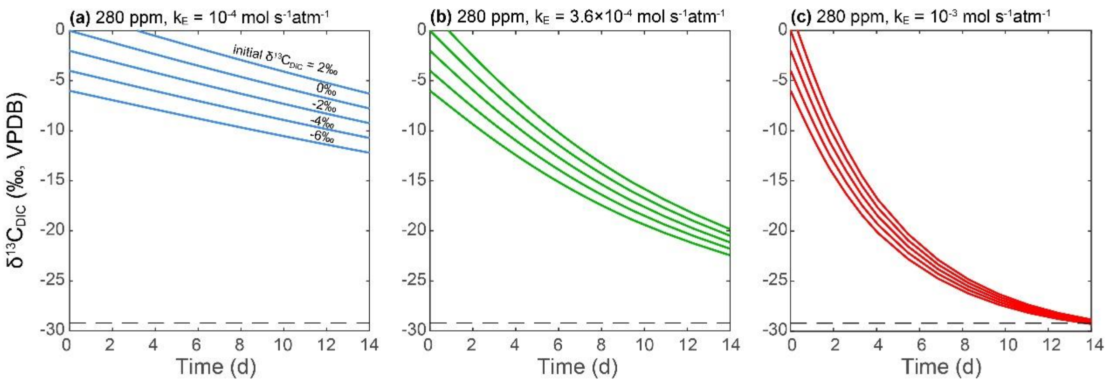

Figure S2. The DIC carbon isotope evolution in the 280 ppm CO_2 treatment with different initial $\delta^{13}C_{DIC}$. (a-c) Simulations for 280 ppm treatment with different exchanging rate constants ranging from 10^{-4} to 10^{-3} mol s^{-1} atm^{-1}.

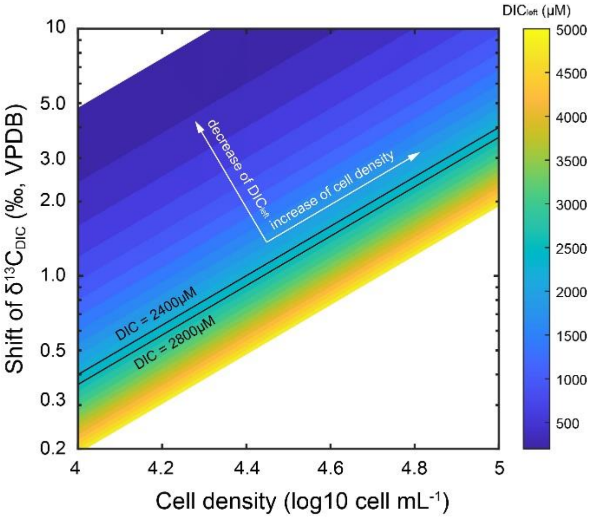

Figure S3. Potential carbon isotope ratio shifts of DIC during culture. The area between the two black lines represents the DIC concentration reported in Liu et al[2]. Please note the log scales for both cell density and carbon isotope ratio axis.

Additional References

1. Van Heuven, S., Pierrot, D., Rae, J., Lewis, E. & Wallace, D. CO2SYS v 1.1, MATLAB program developed for CO2 system calculations. *ORNL/CDIAC-105b. Oak Ridge, TN: Oak Ridge National Laboratory* (2011).
2. Liu, Y. W., Eagle, R. A., Aciego, S. M., Gilmore, R. E. & Ries, J. B. A coastal coccolithophore maintains pH homeostasis and switches carbon sources in response to ocean acidification. *Nat Commun* **9**, 2857, doi:10.1038/s41467-018-04463-7 (2018).
